# *Mycoplasma genitalium*: an efficient strategy to generate genetic variation from a minimal genome

**DOI:** 10.1111/j.1365-2958.2007.05911.x

**Published:** 2007-10

**Authors:** Liang Ma, Jørgen S Jensen, Leann Myers, Judy Burnett, Mary Welch, Qiuyao Jia, David H Martin

**Affiliations:** 1 Section of Infectious Diseases, Department of Medicine, Louisiana State University Health Sciences Center New Orleans, LA 70112, USA; 2 Statens Serum Institute DK2300 Copenhagen S, Denmark; 3 Department of Biostatistics, Tulane University New Orleans, LA 70112, USA

## Abstract

*Mycoplasma genitalium*, a human pathogen associated with sexually transmitted diseases, is unique in that it has smallest genome of any known free-living organism. The goal of this study was to investigate if and how *M. genitalium* uses a minimal genome to generate genetic variations. We analysed the sequence variability of the third gene (MG192 or *mgpC*) of the *M. genitalium* MgPa adhesion operon, demonstrated that the MG192 gene is highly variable among and within *M. genitalium* strains *in vitro* and *in vivo*, and identified MG192 sequence shifts in the course of *in vitro* passage of the G37 type strain and in sequential specimens from an *M. genitalium*-infected patient. In order to establish the origin of the MG192 variants, we examined nine genomic loci containing partial copies of the MgPa operon, known as MgPar sequences. Our analysis suggests that the MG192 sequence variation is achieved by recombination between the MG192 expression site and MgPar sequences via gene cross-over and, possibly, also by gene conversion. It appears plausible that *M. genitalium* has the ability to generate unlimited variants from its minimized genome, which presumably allows the organism to adapt to diverse environments and/or to evade host defences by antigenic variation.

## Introduction

*Mycoplasma genitalium* has been recognized as an important cause of non-gonococcal urethritis in men (Taylor-Robinson, 2002; Jensen, 2006) and is likely to be associated with genital tract inflammatory diseases in women, including cervicitis, endometritis, pelvic inflammatory disease and tubal factor infertility (Moller *et al*., 1984; Clausen *et al*., 2001; Cohen *et al*., 2002; Manhart *et al*., 2003). In addition, there is evidence from at least two studies suggesting that *M. genitalium* is associated with increased risk of HIV-1 (Perez *et al*., 1998; Kapiga *et al*., 2006). A recent molecular study has established that *M. genitalium* is sexually transmitted (Hjorth *et al*., 2006). Like other pathogenic mycoplasmas, *M. genitalium* is capable of causing chronic infections. It has been documented that *M. genitalium* infection can persist for 5 months in animal models (Taylor-Robinson *et al*., 1987) and for more than 2 years in humans (Hjorth *et al*., 2006) although the mechanisms for persistence remain undetermined.

For many bacterial pathogens, antigenic variation of surface-exposed proteins is thought to be an important strategy that allows the organism to persist by adapting to microenvironmental changes and evading host defences (Seifert and So, 1988; van der Woude and Baumler, 2004). Studies of antigenic variation in mycoplasmas have elucidated a variety of molecular mechanisms for generating high-frequency surface protein phase or size variations (Wise, 1993; Citti and Rosengarten, 1997). Well-documented examples include variation of the lipoprotein family in *Mycoplasma hyorhinis* (Rosengarten and Wise, 1990), *M. pulmonis* (Bhugra *et al*., 1995) and *M. bovis* (Lysnyansky *et al*., 1996) and of the haemagglutinin in *M. synoviae* (Noormohammadi *et al*., 2000). Most of these antigenic variations result from DNA rearrangements and are commonly associated with homologous recombination or site-specific recombination (Dybvig and Voelker, 1996; van der Woude and Baumler, 2004). *M. genitalium* has the smallest genome (580 kb) known to date for a self-replicating organism; its 470 genes are organized in a frugal manner with minimal spacer regions between them (Fraser *et al*., 1995). It has been suggested that genome reduction benefits the organism through greater reproductive efficiency in addition to reduction of energy requirements in limited nutrient environments (Maniloff, 1996). With such a minimal genome, the question is how does *M. genitalium* generate sufficient genetic and antigenic variation to adapt to diverse environments and to evade host defences to permit persistence.

The adhesin protein MgPa of *M. genitalium*, corresponding to the adhesin molecule P1 of *Mycoplasma pneumoniae*, is one of the major surface proteins in this organism and has an important role in the attachment of the organism to host epithelial cells (Hu *et al*., 1987). Serological studies have shown that MgPa is the immunodominant protein of *M. genitalium* (Hu *et al*., 1987; Morrison-Plummer *et al*., 1987; Wang *et al*., 1997; Svenstrup *et al*., 2006). The gene encoding MgPa is organized in an operon containing three genes in the order of MG190 (or *mgp*A), MG191 (or *mgp*B) and MG192 (or *mgp*C), with intervening regions of 6 bp and 1 bp respectively (Inamine *et al*., 1989; Fraser *et al*., 1995). It is believed that these three genes are co-transcribed (Inamine *et al*., 1989; Musatovova *et al*., 2003). Molecular studies of *M. genitalium* mutants lacking the MG191 or the MG192 gene have revealed that the encoded proteins are essential for the proper assembly and development of the terminal organelle (Burgos *et al*., 2006). According to the complete genome sequence of the *M. genitalium* type strain G37 (Fraser *et al*., 1995), designated as G37^T^, the expression site for each of these three genes is present in only one copy but there are nine repetitive elements in the form of truncated copies of the MG191 and MG192 genes dispersed throughout the genome, which are designated as MgPa repeats or MgPar sequences (Fraser *et al*., 1995; Iverson-Cabral *et al*., 2006). A few studies have demonstrated that MG191 sequence variation occurs extensively within and among *in vitro* cultured strains and *in vivo* specimens obtained from patients and chimpanzees with chronic *M. genitalium* infection (Peterson *et al*., 1995; Iverson-Cabral *et al*., 2006; Jensen, 2006). Some of these variations could be explained by homologous recombination between the MG191 expression site and MgPar sequences present in the type strain G37^T^. These observations led to the hypothesis that such recombination would generate antigenic variation, allowing *M. genitalium* to evade the host immune response. However, the molecular mechanisms for these proposed recombination events have not been defined. In fact, nothing is known of the MgPar loci of *M. genitalium* strains other than those in the published G37^T^ genome sequence (Fraser *et al*., 1995). Because most MgPars contain not only regions homologous to MG191 but also regions homologous to MG192, it is of particular interest to ask whether MG192 also undergoes genetic and antigenic variation. In order to address this question more information is needed on sequence homology of the MgPars and MG192 as well as the exact location of these homologous regions.

In this article, we describe the number and architecture of MG192 homologous regions in the MgPars, show the degree of sequence variation of MG192 among and within *M. genitalium* strains *in vitro* and *in vivo*, demonstrate MG192 sequence shifts in the type strain G37 during *in vitro* passage as well as in clinical specimens obtained from an *M. genitalium*-infected patient, and report remarkable sequence variation of MgPar regions among *M. genitalium* strains. Furthermore we performed preliminary investigations of the molecular mechanism of recombination between the MG192 expression site and MgPar sequences, and provide evidence that gene cross-over and, possibly, also gene conversion mechanisms are involved in recombination.

## Results

### The homology between the MG192 gene and MgPar sequences in the genome of the *M. genitalium* type strain G37^T^

**Fig. 1:**
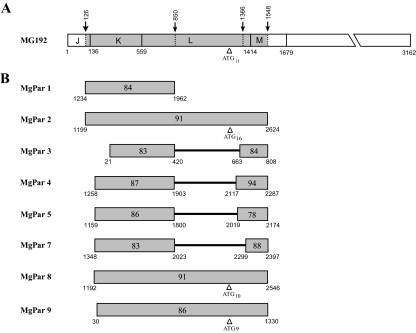
Sequence homology between MG192 and MgPars in *M. genitalium* G37^T^. A. Schematic representation of the structure of the full-length MG192 gene based on the complete genome sequence of *M. genitalium* G37^T^ (GenBank Accession No. NC_000908). The nucleotide positions are in relation to the predicted translational start site of the MG192 gene. Divisions J through M indicated by solid vertical lines represent the restriction fragments described previously (Dallo and Baseman, 1991; Peterson *et al*., 1995). The region highlighted in grey (nt 126–1548) represents the variable region with homology to eight of the nine MgPar sequences shown in (B). This variable region can be further divided into three segments JKL, L and LM (indicated by dotted vertical lines and arrows), which are homologous to different parts of MgPars. Triangle indicates the location of an 11-copy AGT tandem repeat motif (AGT_11_). B. Schematic representation of the nucleotide sequence alignment of eight MgPars from the *M. genitalium* G37^T^ genome. Numbers bordering each shaded area refer to the nucleotide positions in the full-length MgPars as described by Iverson-Cabral *et al*. (2006). Numbers inside the shaded areas indicate the percentage of identity to the corresponding regions in MG192 shown in (A). Triangles indicate the location of the AGT tandem repeat motif with the number of repeat units shown in subscript. Solid black lines in MgPars 3, 4, 5 and 7 represent regions with homology to MG191.

First, we did pairwise comparisons of the G37^T^ MG192 gene with each of the nine MgPars reported by Iverson-Cabral *et al*. (2006) and found three distinct regions in the MG192 gene: the first and third regions spanning from nucleotide (nt) 1–125 and 1549–3162, respectively, are non-repetitive or conserved regions which have no homology to any of the nine MgPar sequences; the second region (nt 126–1548) is variable and has significant homology (78–94% identity) to all nine MgPar sequences except for MgPar 6 (Fig. 1). Based on the restriction maps described by Dallo and Baseman (1991) and Peterson *et al*. (1995), the MG192 variable region can be divided into three divisions: JKL (nt 126–850), L (nt 851–1365) and LM (nt 1366–1548). MgPar 1 contains a 729 bp fragment that is homologous only to the JKL division. MgPars 3, 4, 5 and 7 each contains two regions that are homologous to the JKL and LM divisions and which are separated by a segment with homology to MG191 (see Table S1). MgPars 2, 8 and 9 have segments which are homologous to all three divisions. An AGT tandem repeat motif is present in MG192 homologous regions of MgPars 2, 8 and 9 and in the L division of MG192, with the repeat number varying from 9 to 16. While there has been an alternative assignment (TAG) for the repeat unit of this repeat motif (van Belkum *et al*., 1998; Iverson-Cabral *et al*., 2006) we think that it is more appropriate to assign the repeat unit as AGT as it is the codon for serine based on the MG192 open reading frame (ORF).

### Sequence variation of MG192 among American Type Culture Collection (ATCC) strains of *M. genitalium* and their derivatives

**Fig. 2:**
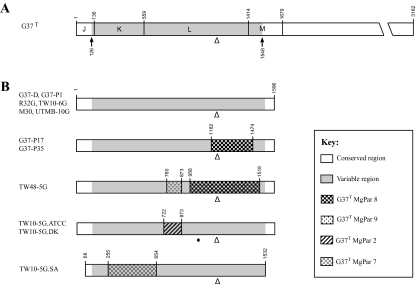
MG192 sequence variation among *M. genitalium* ATCC strains and their derivatives cultured *in vitro*. A. Diagram of the full-length MG192 gene of G37^T^, as shown in Fig. 1. B. Schematic drawing of the MG192 variable regions from *M. genitalium* strains cultured *in vitro* (see Table 1). Regions showing the longest stretch of sequence identity with the MgPars of G37^T^ are indicated by identical patterns, as shown in the key. Numbers bordering each shaded area correspond to the nucleotide positions in the full-length MG192 gene of G37^T^. Triangle indicates the location of the AGT tandem repeats, with detailed information on repeat numbers shown in Table 2. Diamond indicates a single-nucleotide substitution from A to G. The sequences of the MG192 genes for G37-P35, TW48-5G and TW10-5G.ATCC have been submitted to GenBank under Accession No. EF117280, EF117281 and EF117282 respectively.

We examined the MG192 variable region in *M. genitalium* American Type Culture Collection (ATCC) strains and their derivatives (Table 1). A fragment of approximately 1.7 kb covering the entire MG192 variable region was amplified by PCR and sequenced directly and after subcloning. For each strain, we identified a mixture of two or three MG192 sequences that differed from each other only in the number of the AGT tandem repeats (Table 2). Aside from the AGT repeat number variation, the MG192 sequences in all ATCC strains except for TW48-5G and TW10-5G were identical to that of the published G37^T^ genome sequence (Fig. 2). The MG192 sequence of TW48-5G contained a total of 28 nucleotide substitutions and one triplet deletion in the regions flanking the AGT tandem repeats compared with the G37^T^ genome sequence. The MG192 sequence of TW10-5G (designated as TW10-5G.ATCC in Fig. 2) had 67 nucleotide substitutions, three triplet insertions and one triplet deletion upstream of the AGT repeats. These nucleotide substitutions, insertions and deletions did not cause frameshifts or stop codons in the predicted ORF but did code for different amino acid sequences.

**Table 2:** Variable number of AGT tandem repeats in the *M. genitalium* MG192 gene.

Specimen^a^	No. of AGT tandem repeats^b^	No. of total clones analysed
ATCC strains		
G37-D	11_21_, 10_3_ and 12_1_	25
G37-P1	11_8_, 10_4_, 12_2_ and 9_1_	15
TW10-5G.ATCC	11_11_ and 10_1_	12
M30	10_4_ and 9_1_	5
TW10-6G	8_4_ and 9_1_	5
R32G [R32]	12_4_, 11_1_ and 10_1_	6
TW48-5G	11_8_, 10_1_ and 9_1_	10
UMTB-10G	11_2_, 10_2_ and 8_1_	5
ATCC-related strains		
G37-P17	10_3_, 11_1_, 9_1_ and 8_1_	6
G37-P35	10_25_, 9_5_, 11_1_, 8_1_ and 7_1_	33
TW10-5G.DK	11_11_ and 10_1_	12
TW10-5G.SA	11	–c
Patient specimens		
199.0	6_10_	10
199.1	6_18_	18

a Same as Table 1.

b Numbers in subscript indicate the number of plasmid clones analysed for respective repeats, which are listed in the order from high to low frequency.

c Based on the report of Musatovova *et al*. (2006). Not sequenced in this study.

**Table 1:** *M. genitalium* specimens used in this study.

Specimen	Description	Reference
ATCC strains		
G37-D	ATCC33530D, G37 genomic DNA	Tully *et al*. (1981)
G37-P1	ATCC33530 (G37), frozen culture	Tully *et al*. (1981)
TW10-5G.ATCC	ATCC49123, frozen culture	Baseman *et al*. (1988)
M30	ATCC49895, frozen culture	Baseman *et al*. (1988)
TW10–6G	ATCC49896, frozen culture	Baseman *et al*. (1988)
R32G [R32]	ATCC49897, frozen culture	Baseman *et al*. (1988)
TW48-5G	ATCC49898, frozen culture	Baseman *et al*. (1988)
UMTB-10G	ATCC49899, frozen culture	Tully *et al*. (1995)
ATCC-related strains		
G37-P17	G37 (ATCC33530) passage No. 17	This study
G37-P35	G37 (ATCC33530) passage No. 35	This study
TW10-5G.DK	TW10-5G strain obtained from J.G. Tully prior to deposit into ATCC	Jensen (2006)
TW10-5G.SA	TW10-5G strain maintained in the University of Texas Health Sciences Center at San Antonio, Texas	Musatovova *et al*. (2006)
Patient specimens		
199.0	Urine specimen from a New Orleans patient (No. 199) at his first visit on 28 January 2003	Ma and Martin (2004)
199.1	Follow-up urine specimen from the same patient above on 7 February 2003	Ma and Martin (2004)

Surprisingly, the MG192 sequence of TW10-5G.ATCC differed from the recently published sequence for the TW10-5G strain maintained in the University of Texas Health Sciences Center at San Antonio (GenBank Accession No. AY679761, designated here as TW10-5G.SA) (Musatovova *et al*., 2006). We also examined the MG192 sequence of the TW10-5G strain that was provided to one of us (J.S.J.) by J.G. Tully prior to the time that the strain was deposited into the ATCC collection. This strain (designated here as TW10-5G.DK) had been passaged 11 times in Dr Tully's laboratory and subsequently propagated for five passages before being studied as described here. By analysis of the PCR products amplified in three independent experiments using different primers, TW10-5G.ATCC and TW10-5G.DK consistently showed the same MG192 sequence (Fig. 2).

### Detection of MG192 sequence variation in the *M. genitalium* type strain G37 during *in vitro* passage

**Fig. 3:**
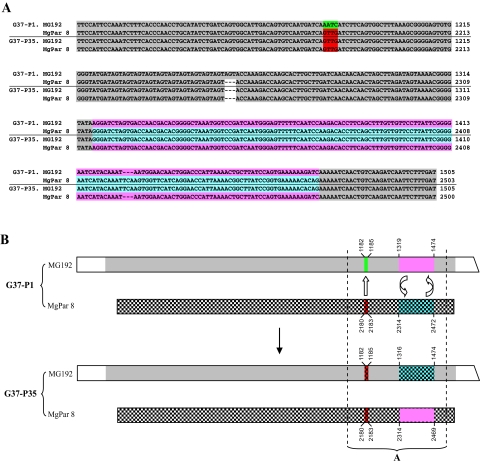
Homologous recombination between MG192 and MgPar 8 in the *M. genitalium* type strain G37 during *in vitro* passage. A. Alignment of a portion of the MG192 variable region and MgPar 8 in G37-P1 and G37-P35 showing the sequence exchange in the recombination site. Identical sequences are highlighted in the same colour. The number of plasmid clones analysed for each locus is listed in Table 3. All clones analysed for each locus showed homogenous sequence except for the AGT short tandem repeat number variation, with only the most common repeat number shown in this alignment. The sequences of the MG192 and MgPar 8 identified in G37-P35 have been submitted to GenBank under Accession No. EF117280 and EF7289 respectively. B. A model of homologous recombination between MG192 and MgPar 8 in the G37 type strain. Nucleotide positions shown are relative to the full-length MG192 or MgPar 8 of G37-P1, both of which are identical to those of G37^T^. Regions containing the longest stretch of identical sequences are indicated by the same shading and colour. The regions between dotted vertical lines (corresponding to the sequences shown in A) indicate where sequence exchanges took place, with reciprocal exchange (gene cross-over) shown by two curved arrows and non-reciprocal exchange (gene conversion) shown by one straight arrow.

We passed the G37 ATCC strain serially 35 times *in vitro* and examined the entire MG192 variable region at three passage levels (designated here as G37-P1, G37-P17 and G37-P35) as well as in the genomic DNA of G37 directly obtained from ATCC (designated here as G37-D). In each passage, sequencing of individual plasmid clones of the PCR products identified a mixture of three to five MG192 sequences that differed from each other only in the number of the AGT tandem repeats (Table 2). Aside from the AGT repeat number variation, the MG192 sequence in G37-D and G37-P1 was identical to that of the published G37^T^ genome sequence, whereas the MG192 sequence in G37-P17 and G37-P35 was identical between them but different from that of G37^T^ (Fig. 2). Compared with the G37^T^ MG192 sequence, G37-P17 and G37-P35 exhibited sequence variation resulting from 16 nucleotide substitutions and one triplet insertion in the regions flanking the AGT repeat motif (Fig. 3A). None of these nucleotide substitutions or insertions introduced frameshifts or stop codons in the predicted ORFs while the deduced amino acid sequence of these variants differed from that of G37^T^ MG192.

**Table 3:** Comparison of MgPars in the *M. gentalium* strains examined in this study with those of G37^T^.

	No. of nucleotides different relative to G37^T^ MgPars^a^								
	
	MgPar 1 1234−1962	MgPar 2^b^ 1199−2624	MgPar 3 21–808	MgPar 4 1258−2287	MgPar 5 1159−2174	MgPar 6 1–385	MgPar 7 1348−2397	MgPar 8^b^ 1192−2546	MgPar 9^b^ 30–1330
G37-P1	–	0	–	–	–	–	–	0 (22)	0
G37-P35	0	0	0	0	0	0	0	18 (30^c^)	0
TW10-5G.ATCC	0	35 (9)	0	0	0	5	0	0	0
TW48-5G	0	0	0	0	1	0	0	43 (10)	0 (10)
199.0	133 (5)	258 (30^c^)	110 (5)	149 (5)	212 (7)	65	142 (7)	207 (10)	223 (8)
199.1	133 (5)	258 (30^c^)	110 (5)	149 (6)	212 (5)	65	142 (7)	207 (8)	223 (9)

a Based on the report of Iverson-Cabral *et al*. (2006). The regions included for comparison are listed under the names of MgPars.

The number of plasmid clones analysed for selected MgPars was given in parenthesis. MgPars not examined in G37-P1 are indicated by ‘–’.

b Variation in the AGT tandem repeat number is not taken into account for the ATCC strains and their derivatives.

c *P* < 0.005 for finding a different sequence based on the exact binomial 95% confidence interval.

### Extensive variation and rapid shift of the MG192 sequences within a single *M. genitalium* strain from an infected patient

**Fig. 4:**
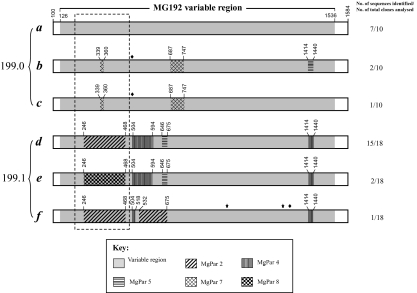
representation of MG192 sequence variation and rapid shift identified in two sequential samples (No. 199.0 and 199.1) obtained 10 days apart from an *M. genitalium*-infected patient. A total of six MG192 variants (*a* to *f*) were identified from these two specimens, with three variants present in each specimen. The sequence of the variant *a*, which is the most predominant variant from the first specimen, is considered the prototype. Various patterns in other five variants represent regions that are different from the variant *a* and instead have homology to MgPars identified in this patient, as indicated by the key. The diamond indicates single-nucleotide substitutions, which can be traced to a specific MgPar sequence, whereas the star indicates single-nucleotide substitutions, which are not present in any other MG192 variants or MgPars. Numbers above each region refer to the nucleotide positions in the full-length MG192 of the variant *a*. Detailed nucleotide sequences for the regions included in the box with dashed lines are given in Fig. 6. The number of plasmid clones analysed for MgPars in the patient specimens is listed in Table 3. All clones analysed for each MgPar showed the same sequence except for the triplet repeat number variation in MgPars 2 and 8. The sequences of MG192 variants *a* to *f* and MgPars 1 through 9 identified from this patient's strain have been submitted to GenBank under Accession No. EF117283 to EF117288 and EF117293 to EF117301 respectively.

To test the hypothesis that MG192 variation occurs *in vivo*, we examined two sequential specimens (199.0 and 199.1) obtained 10 days apart from an *M. genitalium*-infected man (Table 1). These two specimens were previously studied for the variable numbers of tandem repeats (VNTR) in the putative lipoprotein gene, MG309, and single-nucleotide polymorphisms in both the rRNA operon (Ma and Martin, 2004) and the MG191 conserved AB region (Hjorth *et al*., 2006). All of these loci are present in a single copy in the genome (Fraser *et al*., 1995). It has been well documented that molecular typing based on these loci, particularly the MG309 VNTR and the MG191 conserved AB region, provides excellent discriminatory power for unrelated *M. genitalium* strains (Ma and Martin, 2004; Hjorth *et al*., 2006). The two sequential specimens showed identical genotypes at all three loci, thus demonstrating that this patient was infected with a single *M. genitalium* strain. Direct sequencing of the MG192 PCR products from both specimens showed a mixture of two or more different sequences. In order to obtain the individual sequences**,** we studied plasmid clones of the PCR products from two independent assays for each specimen. Three MG192 variant sequences were identified in each specimen (Fig. 4). Remarkably, none of these variant sequences were shared between the two specimens. *In silico* translation analysis showed that all these six variant sequences remained in the correct reading frame and are predicted to encode divergent amino acid sequences. Comparison of the entire variable region sequence (nt 126–1548) of the G37^T^ MG192 gene to the patient MG192 sequences showed a 14–15% difference at the nucleotide level and a 15–17% difference at the deduced amino acid level.

**Fig. 6:**
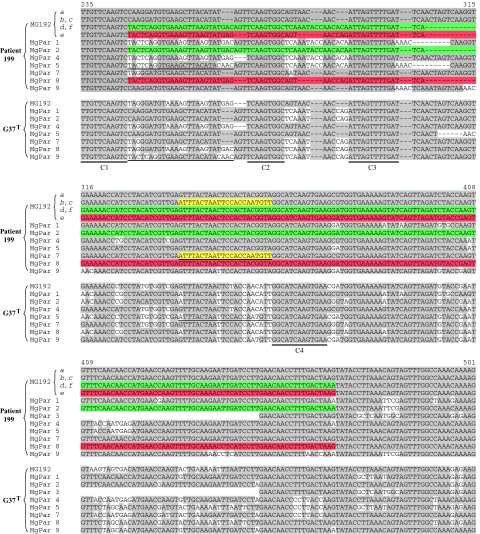
Alignment of a portion of the six MG192 variants (as shown in Fig. 4) and eight MgPar sequences identified in an *M. genitalium*-infected patient 199 and comparison with the MG192 and MgPar sequences of G37^T^. Nucleotide positions are in relation to the predicted translational start site of the full-length G37^T^ MG192 gene. For MgPar 3 in both G37^T^ and the patient's strain, the region preceding the sequence shown has homology to MG191 but not to MG192 (see Fig. 1). Nucleotides identical to the variant *a* sequence are highlighted in grey shading. Three possible recombination events are shown, which involve replacement of part of the MG192 sequence in variant *a* by a segment from MgPar 2 (highlighted in green), MgPar 8 (highlighted in red) and MgPar 7 (highlighted in yellow) respectively. Double underlines C1 to C4 indicate highly conserved regions among all sequences shown. Single underlines indicate identical regions between MgPars in the patient's strain and MgPars in G37^T^. The sequences of MG192 variants *a* to *f* and MgPars 1 through 9 identified from this patient's strain have been submitted to GenBank under Accession No. EF117283 to EF117288 and EF117293 to EF117301 respectively. Alignment of the entire MG192 variable regions and their homologous regions in MgPars shown in this figure is available upon request.

### Sequence variation of the MgPar repeats

To date, there are no published data on MgPar sequences from any strains other than the ATCC G37 type strain. We determined the partial or entire sequences of three MgPars (2, 8 and 9) in G37-P1 and all nine MgPars in G37-P35, TW10-5G.ATCC, TW48-5G, and the two sequential patient specimens (No. 199.0 and 199.1). The results of MgPar sequence variation are summarized in Table 3.

**Fig. 5:**
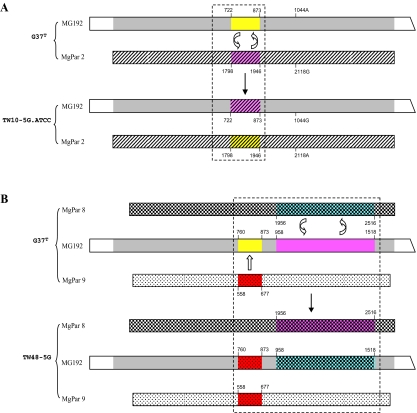
MG192 and MgPar sequence variation in *M. genitalium* ATCC strains TW10-5G and TW48-5G. A. Schematic representation of the MG192 variable region and MgPar 2 in TW10-5G.ATCC compared with those of G37^T^. Numbers below and above each box refer to the nucleotide positions in the full-length MG192 and MgPar 2 of G37^T^. Regions containing identical sequences were indicated by the same shading and colour. The regions included in the box with dashed lines indicate where sequence exchanges potentially took place (detailed sequence alignment for this regions is given in Fig. S1). A reciprocal exchange of a 152 bp fragment is indicated by two curved arrows. Another reciprocal exchange of a single base occurred at nt 1044 of MG192 with nt 2118 of MgPar 2. The MgPar 2 sequence in TW10-5G.ATCC was obtained by sequencing of nine plasmid clones, all of which contained the same sequence except for the expected AGT repeat number variation (from 15 to 17 repeats). The sequences of the MG192 and MgPar 2 in TW10-5G.ATCC have been submitted to GenBank under Accession No. EF117281 and EF117290 respectively. B. Schematic diagram of the MG192 variable region and MgPars 8 and 9 in TW48-5G compared with those of G37^T^. Numbers below and above each box refer to the nucleotide positions in the full-length MG192, MgPar 8 or MgPar 9 of G37^T^. Regions containing identical sequences were indicated by the same shading and colour. The regions included in the box with dashed lines indicate where sequence exchanges took place (detailed sequence alignment for this regions is given in Fig. S2). A non-reciprocal sequence exchange of a 114 bp fragment was indicated by one straight arrow, and a reciprocal sequence exchange of a 561 bp fragment was indicated by two curved arrows. Both MgPars 8 and 9 sequences in TW48-5G were obtained by sequencing of 10 plasmid clones for each MgPar; all clones for each MgPar contained identical sequences except for the expected AGT repeat number variation (9–11 repeats for MgPar 8 and 8–10 repeats for MgPar 9). The sequences of the MG192 and MgPar 8 in TW48-5G have been submitted to GenBank under Accession No. EF117282 and EF117292 respectively.

As expected, the MgPars 2, 8 and 9 in G37-P1 were identical to those of G37^T^ (Iverson-Cabral *et al*., 2006) except for the deletion or insertion of one AGT tandem repeat unit. In G37-P35, MgPar 8 contained a region for which the sequence varied significantly from that of G37^T^ (Fig. 3A) while the other eight MgPars were identical to those of G37^T^ except for the deletion or insertion of one AGT repeat unit in MgPars 2 and 9. We examined 30 plasmid clones of the MgPar 8 PCR product from G37-P35 and found identical sequences in all clones except for variation in the repeat unit number of the AGT tandem repeats. In TW10-5G.ATCC, MgPars 2 and 6 showed sequence variations due to apparent nucleotide substitutions and/or insertions while all other MgPars were identical to the corresponding MgPars of G37^T^ except for the deletion or insertion of one AGT tandem repeat unit in MgPars 8 and 9 (Fig. 5A and Fig. S1). In TW48-5G, MgPar 5 showed only a single base substitution compared with G37^T^ while MgPar 8 had extensive sequence variation in a 561 bp segment (Fig. 5B and Fig. S2). All other TW48-5G MgPars were identical to the corresponding MgPars of G37^T^.

For the two sequential patient specimens 199.0 and 199.1 obtained 10 days apart, we did PCR subcloning and analysed multiple plasmid clones for all of the MgPars except for MgPar 6, which has no identity to MG192 (Table 3). Surprisingly, given the striking sequence variation found in MG192, all clones showed identical sequences for each MgPar between and within the first and second specimen except for the expected tandem repeat variations in MgPar 8. The MgPar 6 sequence obtained by direct sequencing the PCR product was also identical between these two sequential specimens. Of note is the fact that the sequences of all nine patient MgPars differed from those of G37^T^ by 13–20% of nucleotides. Figure 6 shows an alignment of a portion of these MgPar sequences.

### The pattern of MG192 variation *in vitro* is suggestive of DNA recombination with MgPars

To determine the mechanism by which the MgPars recombine into the MG192 expression site, we mapped the regions of sequence exchange in MG192 variantsidentified in this study. As shown in Fig. 3B, by comparative analysis of the MG192 and MgPar sequences in G37-P1 and G37-P35, we observed that the sequence variations noted in MG 192 and MgPar 8 could be accounted for by a reciprocal exchange of a stretch of 155 nucleotides between the MG192 expression site and MgPar 8 as well as a non-reciprocal replacement of four nucleotides in MG192 by the corresponding region in MgPar 8.

Based on exact matches between multiple variable loci inside and outside the MgPa operon, it appears that all ATCC *M. genitalium* strains are very closely related to the G37 type strain and possibly could be the same strain (Kokotovic *et al*., 1999; Ma and Martin, 2004; Hjorth *et al*., 2006; Jensen, 2006). If one considers G37 as the progenitor of TW48-5G, the MG192 sequence variations in TW48-5G can be explained by a non-reciprocal replacement of a 114 bp segment between MG192 and MgPar 9 as well as a reciprocal exchange of a 561 bp segment between MG192 and MgPar 8 (Fig. 5B). Similarly if one considers G37 as the progenitor of TW10-5G.ATCC, the MG192 sequence variations identified in TW10-5G.ATCC can be explained by a reciprocal exchange of a 152 bp fragment between the G37^T^ MG192 expression site and the G37^T^ MgPar 2 and a reciprocal exchange of a single nucleotide located 250 bp downstream of the 152 bp fragment (Fig. 5A and Fig. S1). In fact these two areas are the only known differences in sequence between TW10-5G.ATCC and G37^T^. Similarly the MG192 sequence of TW10-5G.SA could have resulted from a single recombination event involving a 400 bp segment near the 5′ end of the G37^T^ MG192 expression site and the corresponding region of G37^T^ MgPar 7 (Fig. 2). This observation is based on comparison of the published MG192 sequence of TW10-5G.SA (Musatovova *et al*., 2006) with the published MG192 and MgPar sequences of G37^T^ (Fraser *et al*., 1995; Iverson-Cabral *et al*., 2006). Whether this recombination is caused by gene cross-over or gene conversion in this case could not be predicted as the sequences of TW10-5G.SA MgPars have not been published.

### The pattern of MG192 variation *in vivo* is suggestive of DNA recombination with MgPars

To determine if the recombination mechanisms identified *in vitro* as described above also occur *in vivo*, we analysed the MG192 variants and MgPar sequences identified in the two patient specimens obtained 10 days apart. Figure 4 illustrates the structure of the full-length variable region of the six MG192 variants and their homology to the MgPar sequences identified in this patient. Almost all sequence variations seen in each of the MG192 variants can be traced to a specific MgPar donor sequence. In one variant (*f*) there were two single-nucleotide changes which were not present in the corresponding sites of any of the MgPar or MG192 variants. Because the variant sequence *f* was derived from only one clone, it could not be confirmed if these two nucleotide changes are the result of PCR artefacts. Assuming the MG192 variant sequence *a* (identified as the most predominant type from specimen 199.0 obtained at the first visit) is the prototype, the other five MG192 variants appear to have been generated by two to four segmental recombination events between the MG192 expression site and a specific MgPar identified in this patient's *M. genitalium* strain. Given that each MgPar was found to be identical between and within the first- and second-visit specimen, the recombination events all appear to have resulted from duplication of the MgPar sequences, with loss of the segment previously present in the MG192 variable region. Therefore, a non-reciprocal recombination mechanism (gene conversion) may be the mechanism involved in the multiple MG192 recombination events occurring in this patient's *M. genitalium* strain over a relatively short period of 10 days.

### Architecture of the MG192 variable region and the MgPar sequences

Alignment of all MG192 variants and MgPar sequences identified in this study revealed 36 discrete blocks of sequence of 8–43 bp which were completely identical among all MG192 and MgPar sequences examined. These conserved blocks were interspersed between highly variable regions, which differed at both the nucleotide and amino acid levels. Figure 6 shows examples of these constant and variable sequences corresponding to nt 235–501 of the G37^T^ MG192 gene. The conserved sequences could act as anchors for the recombination reactions and/or they could be important in maintaining the structural integrity of the molecule. Detailed comparison of the sequences in the highly variable regions indicates that some elements might be able to translocate from one MgPar to another. For example, a 25 bp element between conserved regions C1 and C2 (Fig. 6), which is unique for MgPar 9 of G37^T^, is present in MgPar 5 rather than MgPar 9 in the patient's strain. Another example is a 22 bp element located between C3 and C4, which is unique for MgPar 5 in G37^T^ but appears to translocate to MgPar 7 in the patient's strain. These observations may suggest that recombination could also occur between two MgPars.

All nine MgPar sequences identified from the patient specimens showed features similar to the G37^T^ MgPars as previously described (Peterson *et al*., 1995; Iverson-Cabral *et al*., 2006; Jensen, 2006), including: the presence of AT-rich sequences, the maintenance of partial ORFs in individual regions with similarity to the MG191 and MG192 sequences, the appearance of stop codons near the beginning and end of the partial ORFs, the occurrence of insertions and deletions in multiples of three nucleotides, and the lack of obvious translational signals. All these features indicate that MgPar sequences are not likely to be directly expressed as functional proteins but rather serve as the donor sequences that recombine into the MG191 and MG192 expression sites. The data presented here strongly support this hypothesis.

## Discussion

This study has demonstrated that, like the MG191 gene, the MG192 gene is highly variable within and among *M. genitalium* strains cultured *in vitro* and from clinical specimens. The variation occurs in the MG192 region (nt 126–1548) that has homology to eight of the nine MgPar loci. To define the molecular mechanisms for the MG192 variation, we have analysed the sequences of the MG192 variable region and all MgPar loci in serial *in vitro* passages of the *M. genitalium* G37 type strain and in sequential specimens from an *M. genitalium*-infected patient. We have found that the MG192 sequence changes rapidly over time *in vitro* and *in vivo*, and provided evidence that the MG192 sequence variation is generated by recombination between the MG192 expression site and MgPar sequences via gene cross-over and, possibly, also by gene conversion. As the MG192 gene is co-transcribed with MG191 (Inamine *et al*., 1989) and both molecules elicit strong immune responses in animal models and in humans during infection (Hu *et al*., 1987; Morrison-Plummer *et al*., 1987; Wang *et al*., 1997; Svenstrup *et al*., 2006), the finding of MG192 sequence variation generated by DNA recombination supports the hypothesis that *M. genitalium* is undergoing antigenic variation in the MgPa operon, which may allow the organism to evade the host immune response and establish persistent infection (Peterson *et al*., 1995; Iverson-Cabral *et al*., 2006; Jensen, 2006).

This study is the first to identify sequence variability of the MG192 and MgPar loci among ATCC *M. genitalium* strains and in clinical specimens, and to demonstrate that MgPar loci serve as the donor sequences that are recombined into the MG192 expression site. The hypothesis of DNA recombination is further supported by the presence in the genome of the basic components required for DNA recombination and repair, including *recA* (MG339), *recU* (MG352), Holliday junction DNA helicases *ruvA* (MG358) and *ruvB* (MG359), formamidopyrimidine-DNA glycosylase *mutM* (MG262.1) and a likely DNA damage-inducible protein gene (MG360) (Fraser *et al*., 1995; Glass *et al*., 2006). Although the homologues of some recombination-related enzymes (such as RecBCD, RecQ, RecO and RecJ) have been described in other organisms, they have not been identified in the *M. genitalium* genome or discovered experimentally. It is likely that the function of such enzymes is performed by other *M. genitalium* enzymes (Fraser *et al*., 1995). For example, the first gene (MG190) in the *M. genitalium* MgPa operon, which is present in single copy in the genome, contains a predicted phosphoesterase motif and has homology to the RecJ enzyme of *Escherichia coli* (Aravind and Koonin, 1998), raising the possibility that MG190 might act as an enzyme involved in homologous recombination (Iverson-Cabral *et al*., 2006).

Our studies suggest that the potential of the MgPa recombination system for generating genetic variation is nearly unlimited. Except for MgPar 6 which has homology only to the MG191 region EF, all MgPars contain 3–5 discrete minicassettes that are homologous to different regions of the MG191 and MG192 expression sites. Studies of the G37 strain maintained in Seattle have shown that sequence variation in the MG191 B, EF and G regions can occur independently (Iverson-Cabral *et al*., 2006). Similarly our studies of *M. genitalium* strains from clinical specimens revealed that recombination in the MG192 JKL and LM regions occurred independently with MgPars (Fig. 4 and L. Ma *et al.*, unpubl. data). If we assume conservatively that each recombination event involves one of the six variable regions in MgPa and its entire corresponding region of one MgPar, the number of potential variants would be 187 500 (see Table S1). However, because chimeric sequences can be generated from small blocks within the donor MgPar sequences, in reality the number of possible combinations would be much greater. In addition, the possibilities exist that recombination can occur between MgPars or between two different variable regions within the MG191 and MG192 expression sites and that other recombination mechanisms such as phase variation (Burgos *et al*., 2006) are also involved in the generation of MG191 and MG192 diversity. All these findings suggest that *M. genitalium* has evolved an efficient recombination system to generate a vast number of variants from a minimal genome.

In the context of the above it should be noted that while comparison of the entire MG192 variable region in G37^T^ and our patient specimens showed great variation at both the nucleotide level (14–15% difference) and the deduced amino acid level (15–17% difference) as would be predicted based on the model we are proposing, the ATCC *M. genitalium* strains were relatively homogenous (Fig. 2). In fact, for four of the ATCC strains the MG192 gene sequences were identical to G37^T^ and in the other two (TW10-5G.ATCC and TW48-5G) the differences could be explained by only one or two recombination events between G37^T^ MG192 and G37^T^ MgPars (Fig. 5). The MgPar sequences from these two strains confirmed that several recombination events indeed may have occurred just as would have been predicted (Fig. 5). In contrast, the MgPar sequences from the patient specimens differed greatly compared with those of G37^T^ (Table 3 and Fig. 6). In our as yet unpublished studies of the MG192 gene and selected MgPar regions of *M. genitalium* specimens obtained from 12 unrelated patients we have confirmed this extraordinary level of variation. These data provide further evidence that the ATCC *M. genitalium* strains are extremely closely related to one another if in fact they actually are not all the same strain (Kokotovic *et al*., 1999; Ma and Martin, 2004; Hjorth *et al*., 2006; Jensen, 2006).

Recombination of surface protein genes to generate genetic and antigenic variation has been described in many other pathogenic microorganisms. The architecture of the MgPa operon in *M. genitalium* is very similar to that of the P1 operon in *M. pneumoniae* (organized as ORF4–P1–ORF6 in which the latter two correspond to MG191 and MG192 respectively), with some portions of the P1 and ORF6 genes repeated seven to nine times throughout the genome (Himmelreich *et al*., 1996). However, in contrast to the MG191 and MG192 genes, the P1 and ORF6 genes show only limited variation (Ruland *et al*., 1994; Kenri *et al*., 1999). While the molecular mechanisms for this variation have not been well defined, preliminary studies suggest that variation in both P1 and ORF6 may be generated by recombination with repetitive elements via a gene conversion mechanism (Ruland *et al*., 1994; Kenri *et al*., 1999). In fact, the gene conversion mechanism has been documented as a common mechanism to generate genetic and antigenic variations in surface structures in other mycoplasma species as well as other sexually transmitted pathogens. The MgPa operon of *M. genitalium* and the P1 operon of *M. pneumoniae* resemble the genetic architecture of the *vlhA* gene of *M. synoviae* (Noormohammadi *et al*., 2000), the pilin gene of *Neisseria gonorrhoeae* (Zhang *et al*., 1992) and the *tprk* gene of *Treponema pallidum* (Centurion-Lara *et al*., 2004), in that all carry a single or no more than two expression sites as well as a repertoire of homologous repeats or pseudogenes that serve as donor sequences and can be recombined into the expression site. However, in contrast to these genes for which the donor sequences are present in tandem arrays, the repetitive elements for the MgPa and P1 operons are dispersed throughout the genome. In all the pathogens mentioned above, studies of the recombination mechanisms have shown a clear bias towards gene conversion, as opposed to gene cross-over (Santoyo and Romero, 2005). This bias has also been demonstrated in studies of the antigenic variation in the *vlsE* gene of *Borrelia burgdorferi* (Zhang and Norris, 1998), the *vmp* gene of *Borrelia hermsii* (Donelson, 1995) and the *msp2* gene of *Anaplasma marginale* (Brayton *et al*., 2001).

In the present study, it appeared that gene cross-over, probably together with gene conversion, occurred *in vitro* in the serial passages of the G37 type strain. The occurrence of the gene cross-over mechanism is strongly supported by the reciprocal exchange of the 150 bp segment between MG192 and MgPar 8 in serial passages of G37-P35 (Fig. 3). The sequence exchange of 4 bp between MG192 and MgPar 8 in the G37-P35 (Fig. 3) likely reflects a non-reciprocal exchange (gene conversion) event as 33 plasmid clones of G37-P35 MG192 and 30 plasmid clones of G37-P35 MgPar 8 were sequenced without a single example of a potential cross-over event at this region (*P*< 0.005 for finding such a clone from the plasmid library, Table 3). However, because the sequence between the 4 bp and 150 bp segment was identical (aside from the AGT tandem repeat number variation as discussed below), an alternative explanation is that two sequential cross-over events had occurred, with the first cross-over event involving both regions and the second cross-over involving the 4 bp region alone. Nonetheless, definitive proof of such events would require single colony cloning studies.

Analysis of the six MG192 variants and nine MgPars in the sequential patient specimens showed that different MgPar segments had recombined into the MG192 expression site without detectable change in MgPar sequences over time, suggesting gene conversion rather than gene cross-over events. If gene cross-over had occurred, we would have expected the MgPar sequences as determined by PCR subcloning analyses to be highly heterogeneous between and/or within the two sequential specimens. For one of the loci where recombination events appeared to have occurred (nucleotides 248–468 in variants *d* and *f*, Fig. 4) 30 MgPar 2 plasmid clones were analysed without a single example of a potential cross-over event, thus supporting gene conversion as the mechanism (*P*< 0.005, Table 3). As discussed above, gene conversion occurs more frequently than gene cross-over in a number of different bacteria and we hypothesize that the same is the case with *M. genitalium*. However, given the strong evidence for the occurrence of gene cross-over in the G37 type strain, it is possible that the patient's strain studied here was unique and that gene cross-over also occurs *in vivo* but could not be distinguished from gene conversion by PCR and sequence analysis of the whole cell population in the specimen as was done here. While single colony cloning would provide the needed definitive evidence for gene conversion as a mechanism of recombination in the *M. genitalium* MG192 gene, this is not possible given the difficulties with culturing the organism (Hamasuna *et al*., 2007). Our hypothesis could be supported through study of more patient specimens and statistical analyses of large samples of plasmid clones as done in the example above.

Despite the strong evidence for the role of homologous recombination in generation of MG192 variation presented in this study, other mechanisms might also be involved. The presence of stretches of conserved sequences within the MG192 variable region (Fig. 6) suggests a possible involvement of a site-specific recombination mechanism. In addition, genetic variation of MG192 also involves gain or loss of AGT tandem repeats (Table 2). In all ATCC *M. genitalium* strains and their derivatives examined in this study, we observed a mixture of two or more AGT repeat variants which differed by only one repeat unit, suggesting that this repeat sequence is undergoing rapid change over time. This observation is further supported by the finding of a significant change in the ratio of populations containing different repeat numbers during serial passage of the G37 type strain *in vitro* (Table 2). Repeat number variation could be generated by recombination between MG192 and MgPars that contain different numbers of these tandem repeats (Fig. 1), but it also could be due to slipped-strand mispairing (Levinson and Gutman, 1987), which may occur alone or together with recombination. The AGT repeat unit encodes serine and changes in the number of repeat units result in heterogeneity in the size of the polyserine tract. The functional significance of the polyserine tract in *M. genitalium* is unknown. Whether it functions as a flexible spacer region to optimize protein interactions, as it has been hypothesized for modular molecules in other organisms (Hall *et al*., 1989; Howard *et al*., 2004), currently cannot be determined.

The occurrence of MG192 sequence variation *in vitro* demonstrates that these events can arise spontaneously, as has been observed in other organisms (Borst, 1991; Criss *et al*., 2005). In *N. gonorrhoeae*, the frequency of gene conversion *in vitro* has been determined to be 3.3 × 10^−2^ per generation (Serkin and Seifert, 1998). In this study we observed change in the G37 MG192 sequence as early as passage 17 but no further change through passage 35. The change may have occurred at a much earlier passage level in response to growth conditions in the laboratory. Perhaps the resulting change in the translated protein conferred some replicative advantage to the organism. The sequence difference found in the TW10-5G strain maintained in different laboratories (TW10-5G.DK and TW10-5G.SA) could have been due to different *in vitro* growth conditions in the two laboratories where they were maintained but this is only speculation. It would be of interest to determine if predictable changes in MG191 and/or MG192 can be induced by differing *in vitro* environments including cultivation with different mammalian cell types. The occurrence of frequent spontaneous variation may be necessary to produce escape variants for selection by the host immune system (Borst, 1991). The striking MG192 variation we found here in patient 199's sequential specimens after an interval of only 10 days may represent an example of this phenomenon. In addition, as both MG192 and MG191 proteins appear to be required for cellular adhesion and terminal organelle development (Burgos *et al*., 2006), variation in the primary amino acid sequence of MG191 and MG192 may change the structure of the MgPa adhesin in response to *in vivo* environmental changes and/or the availability of certain human cell types.

A pragmatic outcome of the present study is the demonstration that inclusion of the MG192 variable region in a genotyping system as recently suggested by Musatovova *et al*. (2006) is inappropriate. Here we have shown that the TW10-5G MG192 sequence published by these investigators (TW10-5G.SA) and the sequence of the ATCC strain (TW10-5G.ATCC) differ significantly. We have shown how this may have occurred by demonstrating sequence shifts of the MG192 variable region between the early and late *in vitro* passages of the G37 strain. Moreover, we have documented dramatic sequence shifts in the MG192 variable region in a human strain over a 10-day period *in vivo*. Musatovova *et al*. (2006) used restriction fragment length polymorphism (RFLP) analysis of a fragment which included parts of the MG192 variable region as the basis for their proposed genotyping system. Over this fragment, there are different numbers of restriction sites between TW10-5G.SA and TW10-5G.ATCC, which likely would have resulted in different RFLP patterns and the false impression that they are two different *M. genitalium* strains. We believe that genotyping should be based on relatively more conserved regions of the *M. genitalium* genome (Ma and Martin, 2004; Hjorth *et al*., 2006).

In summary, we have demonstrated sequence variation of the MG192 and MgPar loci among ATCC *M. genitalium* strains and in clinical specimens, and provided evidence for homologous recombination between the MG192 expression site and MgPars *in vitro* and *in vivo*. Based on preliminary analysis of putative donor sites for MG192 variants, we propose a model of recombination involving gene cross-over and, possibly also gene conversion. As the free-living organism with the smallest known genome, *M. genitalium* appears to have evolved an efficient system to generate a vast number of variants, thus enhancing the organism's survival in differing host environments and allowing it to escape from host defences.

## Experimental procedures

### *Mycoplasma genitalium* specimens

We obtained seven *M. genitalium* strains as freeze-dried culture and/or genomic DNA from ATCC. Detailed information about these specimens is given in Table 1. One vial of each freeze-dried ATCC strain was rehydrated in 0.5 ml of Spiroplasma medium SP-4 and then inoculated into a culture flask containing 10 ml of SP-4. The flask was incubated at 37°C in 5% CO_2_. When the colour of the SP-4 medium changed from red to orange, the culture was collected and concentrated by centrifugation at 9000 *g* for 30 min. One aliquot of the cell pellet for each strain was used for DNA analysis. This material was designated as passage 1 for each strain. For the G37 type strain (ATCC33530), one aliquot of the passage 1 cell pellet (G37-P1) was used to inoculate another flask with 25 ml of fresh SP-4 medium to make passage 2. This procedure was repeated until the passage 35 (G37-P35) was obtained. In addition to the ATCC strains, we studied the TW10-5G strain which was provided to one of us (J.S.J.) by J.G. Tully at the passage level 11 prior to deposit into ATCC (designated as TW10-5G.DK, Table 1). The strain had been passaged further five times before being studied for DNA sequences. Two sequential urine specimens were obtained 10 days apart from a man in New Orleans with acute urethritis (Mena *et al*., 2002). Both samples were positive for *M. genitalium* by our PCR assay and had been genotyped using multiple genomic loci (Ma and Martin, 2004; Hjorth *et al*., 2006). Informed consent was obtained from this patient and the study protocol was approved by the LSUHSC Institutional Review Board.

### PCR and sequencing of the MG192 gene

Based on an alignment of the published MgPa operon and MgPar sequences of G37^T^ (Fraser *et al*., 1995; Iverson-Cabral *et al*., 2006), we designed primers with no homology to any of the MgPar sequences so that only the MG192 gene would be amplified. The sequences of the primers are listed in Table 4. In addition, the previously described primer MG192A (Musatovova *et al*., 2006) was also used.

**Table 4:** Primers used to amplify the *M. genitalium* MG192 and MgPar sequences.

Primer name	Sequence (5′→3′)	Target (sequence location^a^)
5346F	TACCCCAACCAAACCAACTC	MG192 (225850–225869)
227567R	CTTCGCAATTCATTAGGGGAGACA	MG192 (227546–227569)
227529R	GATCTGATCAGTTCTGGAAGGTAAACG	MG192 (227505–227531)
MG192A^b^	CACTAGCCAATACCTTCCTTGTCAAAGAGG	MG192 (225974–226003)
1F2	GAATGGCGGTTTTTACCCAG	MgPar 1 (86583–86602)
1R1	AATTTCACACCAACAATGCC	MgPar 1 (88396–88415)
124F3	TCAGTTTATTGGTCAAAGAAGTCGC	MgPars 1, 2 and 4 (168272–168296)
2F1	CAAGGGGTTGTTTCTTTGC	MgPar 2 (166960–166978)
2R1	GTTCTAGGATCAATTCTTGCAAA	MgPar 2 (168687–168709)
170116R	TACTCACCCGTTCGCCTCTA	MgPar 2 (170095–170114)
3F3	CCAGCAGTTAAGCACAGTGG	MgPar 3 (174730–174749)
3R4	CTTTCACTACCTGTTTCACG	MgPar 3 (176120–176139)
4F3	GGTCAAAGAAGTCGCAAATTTTCTTA	MgPar 4 (214652–214677)
4R1	ATGCATGCAGTGGCTATGG	MgPar 4 (216074–216092)
5F4	TGGCGGTTTTTATCAACTAAATAAAAAC	MgPar 5 (230434–230461)
5R2	GCGGTTTTAATCTTTTGGAG	MgPar 5 (232126–232145)
6F1	TGCAGCCATTTCTTTGATCC	MgPar 6 (273024–273043)
6R1	CATCTGGTAACCAAAGACAC	MgPar 6 (273872–273891)
7F2	TGCTGCGGTGAATTCTAAGG	MgPar 7 (312727–312746)
7F3	TTAATTAATAAAACCTCGACCC	MgPar 7 (314133–314154)
7R1	CAGCGCCAAAAAGATTAGGA	MgPar 7 (315817–315836)
7R3	ATGGATGGTTTGGTCAGCG	MgPar 7 and MG192 (314170–314188)
8F1	GGTAAGCCAATCCGATATTGAG	MgPar 8 (348804–348825)
8F4	ATGGTGGTTTTTATCAACTAAATAAAAAAC	MgPar 8 (350254–350283)
8R2	AATAAAGGTAAAGCCTAGTG	MgPars 5 and 8 (350377–350396)
351964R	CAATCAACTACTTTTCACTTTACACG	MgPar 8 (351940–351965)
9F1	CTGAAAACTAGCGACTGCATTTAG	MgPar 9 (427858–427881)
9F3	GGTTTGTTGACATAAACACTCC	MgPar 9 (428248–428269)
9R1	TTAAGTGCTGCATCCTTCCA	MgPar 9 (430749–430768)
429679R	TTGAATCCTTCACCGGACTATC	MgPar 9 (213693–213714)

a Location of the primer on the *M. genitalium* G37^T^ genome sequence under GenBank Accession No. NC_000908.

b From reference (Musatovova *et al*., 2006).

#### 

##### *M. genitalium* ATCC strains and their derivatives

The DNAs from all *M. genitalium* ATCC strains and their derivatives were amplified using 5346F or MG192A as the forward primer and 227567R as the reverse primer. A single round of PCR amplification was performed with high-fidelity *Pfu* DNA polymerase (Stratagene) and the following cycling conditions: 95°C for 1 min, 35 cycles of 94°C for 45 s, 50°C for 1 min, and 72°C for 3 min.

##### Patient specimens

To amplify the DNAs from patient specimens, we carried out a nested PCR strategy using primers 5346F and 227567R for the first round of amplification and primers MG192A plus 227529R for the second round. The first round of amplification was performed with AmpliTaq Gold DNA polymerase (Applied Biosystems) and a touch-down protocol as described previously (Ma *et al*., 2003). The second round of amplification was performed with high-fidelity *Pfu* DNA polymerase (Stratagene) and the same conditions described above.

Each specimen was amplified by at least two independent PCR assays. All PCR products were initially directly sequenced and then by sequencing of individual plasmid clones after subcloning. The PCR-Script® Amp Cloning Kit (Stratagene) and the TOPO TA Cloning kit (Invitrogen) were used according to manufacturers' standard protocols. There was no difference in the overall distribution of the MG192 sequences between different PCR runs for the same specimens (Fig. 4). All MG192 sequences obtained contained a constant region of 26–125 bp in the 5′ end and of 49 bp in the 3′ end, which were identical to the corresponding region of the G37^T^ MG192 gene. This finding confirmed that the 5′ and 3′ ends of MG192 are highly conserved and all MG192 sequences we obtained were from the MG192 gene; not from any of the MgPar regions.

## PCR and sequencing of the MgPar sequences

The primers used to amplify MgPars were designed from the published genome sequence of the *M. genitalium* G37 strain (Fraser *et al*., 1995) and are listed in Table 4. In general, primers for each MgPar were chosen from regions that have no homology to MG192 or other MgPars in order to assure the specificity. There are three primers 124F3, 7R3 and 8R2 that are shared among MgPars 1, 2 and 4, between MgPar 7 and MG192, and between MgPars 5 and 8 respectively. These were used in combination with another primer specific for the MgPar to be evaluated. We amplified three MgPars (2, 8 and 9) in G37-P1, all nine MgPars in G37-P35, TW10-5G.ATCC, TW48-5G, and the two sequential patient specimens (No. 199.0 and 199.1). MgPars 3 and 6 were amplified in their full length by using primer sets 3F3–3R4 and 6F1–6R1 respectively. For all other MgPars, only the regions that were homologous to the MG192 gene were amplified unless otherwise stated below. The primer sets for MgPars 1, 2, 4, 5, 7, 8 and 9 were 1F2–1R1, 124F3–170116R, 4F3–4R1, 5F4–5R2, 7F3–7R1, 8F4–351964R and 9F1–429679R respectively. The full-length MgPar 2 in TW10-5G.ATCC was amplified in two overlapping fragments using primer sets 2F1–2R1 and 124F3–170116R respectively. The full-length MgPar 7 in TW10-5G.ATCC was amplified in two overlapping fragments using primer sets 7F2–7R3 and 7F3–7R1 respectively. The full-length MgPar 8 in G37-P1, G37-P35, TW48-5G and two patient specimens was amplified in two overlapping fragments using primer sets 8F1–8R2 and 8F4–351964R respectively. The full-length MgPar 9 in TW10-5G.ATCC and TW48-5G was amplified by a single round of PCR using the primer set 9F3–9R1. All amplifications were performed with AmpliTaq Gold DNA polymerase (Applied Biosystems) and a touch-down protocol as described previously (Ma *et al*., 2003). Initially the PCR products of all MgPars were directly sequenced and none of them except MgPars 2, 8 and 9 displayed a mixture of two or more sequences. These sequence mixtures were completely accounted for by variation in the number of AGT repeats; the sequences flanking the repeat region were entirely uniform. However, because the MG192 variable region in the clinical specimens was found to contain sequence mixtures, we further investigated the possibility of existence of sequence mixtures in MgPars by performing subcloning of the PCR products from MgPar 8 in G37-P1 and G37-P35, MgPars 8 and 9 in TW48-5G, MgPars 2 and 8 in TW10-5G, and all MgPars except for MgPar 6 in the two patient specimens. For each PCR product, 5–30 plasmid clones were selected for sequencing. All cloned PCR products showed homogenous sequences within each strain except for the VNTR in MgPar 8 and MgPar 9 as well as the presence of a few scattered single-base substitutions in a few clones, which may represent PCR artefacts.

## DNA and predicted protein sequence analysis

DNA sequencing was carried out by use of an ABI PRISM 3100 automated capillary sequencer (Applied Biosystems). Sequence analysis was performed using the CLC Combined Workbench 2.0 (CLC bio, Aarhus C, Denmark) and the MultAlin software available at http://bioinfo.genopole-toulouse.prd.fr/multalin/ (Corpet, 1988). Representative nucleotide sequences obtained in this study have been submitted to the GenBank database under Accession No. EF117280 to EF117301.

## Statistical analyses

Calculation of the probability of undetected cross-over events as the explanation of sequence data which uniformly show evidence of a conversion event was performed by calculating the exact binomial 95% confidence interval centred on zero for the proportion of cross-over sequences among a set of n sequences all of which were consistent with conversion events. Then the probability of finding a cross-over event if *n* + 1 plasmid clones had been sequenced was calculated.

## Supplementary material

This material is available as part of the online article from: http://www.blackwell-synergy.com/doi/abs/10.1111/j.1365-2958.2007.05911.x (This link will take you to the article abstract).
