# Supplementary material for: Mycoplasma genitalium: an efficient strategy to generate genetic variation from a minimal genome
Source: Mol Microbiol. 2007 Oct;66(1):220–36. doi: 10.1111/j.1365-2958.2007.05911.x (PMC2169797; doi:10.1111/j.1365-2958.2007.05911.x)
Supplement: Supplementary file 1 [file mmi0066-0220-SD1.pdf]

***Supplementary material,* Table S1.** Location and homology of MgPars[a] in relation to MG191 and MG192 based on the *M. genitalium* G37[T] genome.

	MG191 variable regions[a]			MG192 variable regions[b]			No. of regions in MgPar homologous to MG191 and MG192[c]
	B (575-1016)	EF (2292-2876)	G (3305-3550)	JKL (126-850)	L (851-1365)	LM (1366-1548)	
MgPar 1	12-445	445-1038	2010-2170	1234-1962			4
MgPar 2	1-430	430-993		1199-1923	1924-2453	2454-2624	5
MgPar 3			451-664	21-420		663-808	3
MgPar 4	13-442	447-988	1946-2117	1258-1903		2117-2287	5
MgPar 5	13-422	422-994	1843-2017	1159-1800		2019-2174	5
MgPar 6			16-385				1
MgPar 7	11-438	438-1013	2049-2293	1348-2023		2299-2397	5
MgPar 8	10-442	442-1026		1192-1848	1849-2360	2361-2546	5
MgPar 9	1328-1668	1758-2272		30-654	655-1163	1164-1330	5

a. Based on the report of Iverson-Cabral *et al*. (2006). Numbers in parentheses for each region refer to the nucleotide positions relative to the presumed MG191 translational start site.

b. Numbers in parentheses refer to the nucleotide positions relative to the presumed MG192 translational start site.

c. Assuming each individual region is entirely recombined into one site of MgPa, the total number of potential combinations in MgPa would be 187,500 (4 x 5 x 3 x 5 x 5 x 1 x 5 x 5 x 5).

```
G37T        MG192    ACTCACTACCGGCAATGCATATAGAAAACTAAGTGAGTCCTGACCAATTTATGAACCAATTGATGGGACCAAGCAGGGCAAAGGGAAGGATAGTA   712
            MgPar 2  ACTCACTACCGGCAATGCGTATAGAAAATTGGATCAATCCTGACCAATTTACCAACCAATTGATGGGACCAAGCAGGGCAAAGGGAAGGATAGTA  1788
TW10-5G.    MG192    ACTCACTACCGGCAATGCATATAGAAAACTAAGTGAGTCCTGACCAATTTATGAACCAATTGATGGGACCAAGCAGGGCAAAGGGAAGGATAGTA   712
ATCC        MgPar 2  ACTCACTACCGGCAATGCGTATAGAAAATTGGATCAATCCTGACCAATTTACCAACCAATTGATGGGACCAAGCAGGGCAAAGGGAAGGATAGTA  1798

G37T        MG192    GTGGGTGGAGTTCAACTGAAGAAAACGAAGCTAAAAATGATGCGCCCAGTGTTTCTGGAGGGGGATCATCTTCTGGAACATTTAATAAATACCTC   807
            MgPar 2  GTGGGTGGAAT---AGTGAAGAAAACGAAGCTAAAAGTGATGCGCCCCTAAGTACAGGAGGGGGTGCTTCTTCTGGAACATTTAATAAATACCTC  1880
TW10-5G.    MG192    GTGGGTGGAAT---AGTGAAGAAAACGAAGCTAAAAGTGATGCGCCCCTAAGTACAGGAGGGGGTGCTTCTTCTGGAACATTTAATAAATACCTC   804
ATCC        MgPar 2  GTGGGTGGAGTTCAACTGAAGAAAACGAAGCTAAAAATGATGCGCCCAGTGTTTCTGGAGGGGGATCATCTTCTGGAACATTTAATAAATACCTC  1893

G37T        MG192    AACACCAAGCAAGCGTTAGAGAGCATCGGTATCTTGTTTGATGATCAAACCCCAAGAAATGTTATCACCCAACTCTATTATGCTTCTACTAGCAA   902
            MgPar 2  AACACCAAGCAAGCGTTAGAGAGCATCGGCATCTTGTTTGATGGGGATGGAATGAGGAATGTGGTTACCCAACTCTATTATGCTTCTACCAGCAA  1975
TW10-5G.    MG192    AACACCAAGCAAGCGTTAGAGAGCATCGGCATCTTGTTTGATGGGGATGGAATGAGGAATGTGGTTACCCAACTCTATTATGCTTCTACTAGCAA   899
ATCC        MgPar 2  AACACCAAGCAAGCGTTAGAGAGCATCGGTATCTTGTTTGATGATCAAACCCCAAGAAATGTTATCACCCAACTCTATTATGCTTCTACCAGCAA  1988
```

***Supplementary material*, Fig. S1.** Alignment of a portion of the MG192 variable region and MgPar 2 in TW10-5G.ATCC compared to those of G37^T, showing the sequence exchange between MG192 and MgPar 2 in the regions indicated in the box with dashed lines in Fig. 5A. Colors correspond to those in Fig. 5A. Identical sequences are highlighted in same color. Hyphens represent gaps introduced to optimize alignment.

```
G37T   MG192    CTAAAAATGATGCGCCCAGTGTTTCTGGAGGGGGATCATCT------TCTGGAACATTTAATAAATACCTCAACACCAAGCAAGCGTTAG  826
       MgPar 8  CAAAAAATGATGCGCCCAGTGTTTCTGGAGGGGGATCA---GACACCACTTCAAAATTTAAAAGTTACCTCAACACCAAGCAAGCGTTAG 1824
       MgPar 9  CTAAAAATGATGCGCCCCTAAGTACAGGAGGGGGAACATCAGACAACGCTTCAAAATTCACCAAGTACCTCAACACCAAACAAGCATTGG  630
TW48-5G MG192   CTAAAAATGATGCGCCCCTAAGTACAGGAGGGGGAACATCAGACAACGCTTCAAAATTCACCAAGTACCTCAACACCAAACAAGCATTGG  826
       MgPar 8  CAAAAAATGATGCGCCCAGTGTTTCTGGAGGGGGATCA---GACACCACTTCAAAATTTAAAAGTTACCTCAACACCAAGCAAGCGTTAG 1824
       MgPar 9  CTAAAAATGATGCGCCCCTAAGTACAGGAGGGGGAACATCAGACAACGCTTCAAAATTCACCAAGTACCTCAACACCAAACAAGCATTGG  630

G37T   MG192    AGAGCATCGGTATCTTGTTTGATGATCAAACCCCAAGAAATGTTATCACCCAACTCTATTATGCTTCTACTAGCAAGCTAGCAGTCACCA  916
       MgPar 8  AGAGCATCGGCATCTTGTTTGATGGGGATGGAATGAGGAATGTGGTTACCCAGCTCTATTATGCTTCTACTAGCAAGCTAGCAGTCACCA 1914
       MgPar 9  AAAGGATCGGTATCTTGTTTGATGGGGATGGAATGAGGAATGTGGTTACCCAACTCTATTATGCTTCTACTAGCAAGCTAGCAGTCACCA  720
TW48-5G MG192   AAAGGATCGGTATCTTGTTTGATGGGGATGGAATGAGGAATGTGGTTACCCAACTCTATTATGCTTCTACTAGCAAGCTAGCAGTCACCA  916
       MgPar 8  AGAGCATCGGCATCTTGTTTGATGGGGATGGAATGAGGAATGTTATCACCCAACTCTATTATGCTTCTACTAGCAAGCTAGCAGTCACCA 1914
       MgPar 9  AAAGGATCGGTATCTTGTTTGATGGGGATGGAATGAGGAATGTGGTTACCCAACTCTATTATGCTTCTACTAGCAAGCTAGCAGTCACCA  720

G37T   MG192    ACAACCACATTGTCGTGATGGGTAACAGCTTTCTACCCAGCATGTGGTACTGGGTGGTGGAGCGGAGTGCACAGGAAAATGCAAGTAACA 1006
       MgPar 8  ACAACCACATTGTCGTGATGGGTAACAGCTTTCTACCCAGCTTGTGGTACTGGGTGGTGGAGCGGAGTGCAACAACTGATTCATCATCAA 2004
       MgPar 9  ACAACCACATTGTCGTGATGGGTAACAGCTTTCTACCCAGCATGTGGTACTGGGTGGTGGAGCGGAGTGCAACAACTGATTCATCATCAA  810
TW48-5G MG192   ACAACCACATTGTCGTGATGGGTAACAGCTTTCTACCCAGCTTGTGGTACTGGGTGGTGGAGCGGAGTGCAACAACTGATTCATCATCAA 1006
       MgPar 8  ACAACCACATTGTCGTGATGGGTAACAGCTTTCTACCCAGCATGTGGTACTGGGTGGTGGAGCGGAGTGCACAGGAAAATGCAAGTAACA 2004
       MgPar 9  ACAACCACATTGTCGTGATGGGTAACAGCTTTCTACCCAGCATGTGGTACTGGGTGGTGGAGCGGAGTGCAACAACTGATTCATCATCAA  810

G37T   MG192    AACCCACCTGGTTTGCTAATACCAATTTAGACTGAGGAGAAGACAAACAAAAACAATTTGTTGAGAACCAGTTGGGGTATAAGGAAACTA 1096
       MgPar 8  AACCCACCTGGTTTGCTAATACCAATTTAAACTGAGGGGAAGATAAACAAAAACAATTTGTTGAGAACCAGTTGGGGTATAAGGAAACTA 2094
       MgPar 9  AACCCACCTGGTTTGCTAACACCAATTTAGACTGAGGGGAAGACAAACAAAAACAATTTGTAGAGAACCAGTTGGGGTATAAGGAAACTA  900
TW48-5G MG192   AACCCACCTGGTTTGCTAATACCAATTTAAACTGAGGGGAAGATAAACAAAAACAATTTGTTGAGAACCAGTTGGGGTATAAGGAAACTA 1096
       MgPar 8  AACCCACCTGGTTTGCTAATACCAATTTAGACTGAGGAGAAGACAAACAAAAACAATTTGTTGAGAACCAGTTGGGGTATAAGGAAACTA 2094
       MgPar 9  AACCCACCTGGTTTGCTAACACCAATTTAGACTGAGGGGAAGACAAACAAAAACAATTTGTAGAGAACCAGTTGGGGTATAAGGAAACTA  900

G37T   MG192    CCAGTACCAATTCCCACAACTTCCATTCCAAATCTTTCACCCAACCTGCATATCTGATCAGTGGCATTGACAGTGTCAATGATCAAATCA 1186
       MgPar 8  CCAGTACCAATTCCCACAACTTCCATTCCAAATCTTTCACCCAACCTGCATATCTGATCAGTGGCATTGACAGTGTCAATGATCAGTTGA 2184
       MgPar 9  CCAGTACCAATTCCCACAACTTCCATTCCAAATCTTTCACCCAACCTGCATATCTGATCAGTGGCATTGACAGTGTCAATGATCAAATCA  990
TW48-5G MG192   CCAGTACCAATTCCCACAACTTCCATTCCAAATCTTTCACCCAACCTGCATATCTGATCAGTGGCATTGACAGTGTCAATGATCAGTTGA 1186
       MgPar 8  CCAGTACCAATTCCCACAACTTCCATTCCAAATCTTTCACCCAACCTGCATATCTGATCAGTGGCATTGACAGTGTCAATGATCAAATCA 2184
       MgPar 9  CCAGTACCAATTCCCACAACTTCCATTCCAAATCTTTCACCCAACCTGCATATCTGATCAGTGGCATTGACAGTGTCAATGATCAAATCA  990

G37T   MG192    TCTTCAGTGGCTTTAAAGCGGGGAGTGTGGGGTATGATAGTAGTAGTAGTAGTAGTAGTAGTAGTAGTAGTACCAAAGACCAAGCACTTG 1276
       MgPar 8  TCTTCAGTGGCTTTAAAGCGGGGAGTGTGGGGTATGATAGTAGTAGTAGTAGTAGTAGTAGTAGT---ACCAAAGACCAAGCACTTG 2271
       MgPar 9  TCTTCAGTGGCTTTAAAGCGGGGAGTGTGGGGTATGATAGTAGTAGTAGTAGTAGTAGTAGT------ACCAAAGACCAAGCACTTG 1074
TW48-5G MG192   TCTTCAGTGGCTTTAAAGCGGGGAGTGTGGGGTATGATAGTAGTAGTAGTAGTAGTAGTAGTAGTAGTAGTACCAAAGACCAAGCACTTG 1276
       MgPar 8  TCTTCAGTGGCTTTAAAGCGGGGAGTGTGGGGTATGATAGTAGTAGTAGTAGTAGTAGTAGTAGT---ACCAAAGACCAAGCACTTG 2271
       MgPar 9  TCTTCAGTGGCTTTAAAGCGGGGAGTGTGGGGTATGATAGTAGTAGTAGTAGTAGTAGTAGT------ACCAAAGACCAAGCACTTG 1074

G37T   MG192    CTTGATCAACAACAACTAGCTTAGATAGTAAAACGGGGTATAAGGATCTAGTGACCAACGACACGGGGCTAAATGGTCCGATCAATGGGA 1366
       MgPar 8  CTTGATCAACAACAACTAGCTTAGATAGTAAAACGGGGTATAGGGATCTAGTGACCAACGACACGGGGCTAAATGGTCCGATCAATGGGA 2361
       MgPar 9  CTTGATCAACAACAACTAGCTTAGATAGTAAAACGGGGTATAAGGATTTGGTGACCAACGACACGGGATTAAATGGTCCAATCAATGGGA 1164
TW48-5G MG192   CTTGATCAACAACAACTAGCTTAGATAGTAAAACGGGGTATAAGGATTTGGTGACCAACGACACGGGGCTAAATGGTCCGATCAATGGGA 1366
       MgPar 8  CTTGATCAACAACAACTAGCTTAGATAGTAAAACGGGGTATAGGGATCTAGTGACCAACGACACGGGGCTAAATGGTCCGATCAATGGGA 2361
       MgPar 9  CTTGATCAACAACAACTAGCTTAGATAGTAAAACGGGGTATAAGGATTTGGTGACCAACGACACGGGATTAAATGGTCCAATCAATGGGA 1164

G37T   MG192    GTTTTTCAATCCAAGACACCTTCAGCTTTGTTGTTCCTTATTCGGGGAATCATACAAAT---AATGGAACAACTGGACCCATTAAAACTG 1453
       MgPar 8  GTTTTTCAATCCAAGACACCTTCAGCTTTGTTGTTCCTTATTCGGGGAATCATACAAATTCAAGTGGTTCATCAGGAACCATTAAAACGG 2451
       MgPar 9  GTTTTTCAATCCAAGACACCTTCTCATTCGTTGTTCCTTATTCGGGGAATCATAGTAAT---CAAATTTCATCAGGAACCATTAAAACTG 1254
TW48-5G MG192   GTTTTTCAATCCAAGACACCTTCAGCTTTGTTGTTCCTTATTCGGGGAATCATACAAATTCAAGTGGTTCATCAGGAACCATTAAAACGG 1453
       MgPar 8  GTTTTTCAATCCAAGACACCTTCAGCTTTGTTGTTCCTTATTCGGGGAATCATACAAAT---AATGGAACAACTGGACCCATTAAAACTG 2451
       MgPar 9  GTTTTTCAATCCAAGACACCTTCTCATTCGTTGTTCCTTATTCGGGGAATCATAGTAAT---CAAATTTCATCAGGAACCATTAAAACTG 1254

G37T   MG192    CTTATCCAGTGAAAAAAGATCAAAAATCAACTGTCAAGATCAATTCTTTGATTAACGCTACGCCCTTGAATAGTTATGGGGATGAGGGGA 1543
       MgPar 8  CTTATCCGGTGAAAAACACAGAAAAATCAACTGTCAAGATCAATTCTTTGATCAACGCTACGCCGTTGAATAGTTATGGGGATGAGGGGA 2541
       MgPar 9  CTTATCCAGTGAAAAAAGATGAAGCTTCCCAAGTAGCGATCAATTCCTTGAATCAACCTACGCCGTTGAATAGTTATGG            1333
TW48-5G MG192   CTTATCCGGTGAAAAACACAGAAAAATCAACTGTCAAGATCAATTCTTTGATCAACGCTACGCCGTTGAATAGTTATGGGGATGAGGGGA 1543
       MgPar 8  CTTATCCAGTGAAAAAAGATCAAAAATCAACTGTCAAGATCAATTCTTTGATTAACGCTACGCCCTTGAATAGTTATGGGGATGAGGGGA 2541
       MgPar 9  CTTATCCAGTGAAAAAAGATGAAGCTTCCCAAGTAGCGATCAATTCCTTGAATCAACCTACGCCGTTGAATAGTTATGG             133
```

***Supplementary material*, Fig. S2.** Alignment of a portion of the MG192 variable region and MgPars 8 and 9 in TW48-5G compared to those of G37^T, showing the sequence exchange between MG192 and MgPar 8 or 9 in the regions indicated in the box with dashed lines in Fig. 5B. Colors correspond to those in Fig. 5B. Nucleotide sequences identical to those of G37^T MG192 are highlighted in gray shading. The regions highlighted in red represent a non-reciprocal exchange event between MG192 and MgPar 9, whereas the regions highlighted in purple and turquoise represent a reciprocal exchange event between MG192 and MgPar 8.
